# The coordination of major events in C_4_ photosynthesis evolution in the genus *Flaveria*

**DOI:** 10.1038/s41598-021-93381-8

**Published:** 2021-08-02

**Authors:** Ming-Ju Amy Lyu, Udo Gowik, Steve Kelly, Sarah Covshoff, Julian M. Hibberd, Rowan F. Sage, Martha Ludwig, Gane Ka-Shu Wong, Peter Westhoff, Xin-Guang Zhu

**Affiliations:** 1 National Key Laboratory of Plant Molecular Genetics, CAS Center for Excellence in Molecular Plant Sciences, Institute of Plant Physiology and Ecology, Chinese Academy of Sciences, Shanghai, China; 2 Institute of Plant Molecular and Developmental Biology, Heinrich-Heine-University, Dusseldorf, Germany; 3 Department of Plant Sciences, University of Oxford, Oxford, UK; 4 Department of Plant Sciences, University of Cambridge, Cambridge, UK; 5 School of Molecular Sciences, University of Western Australia, Crawley, WA Australia; 6 Department of Ecology and Evolutionary Biology, University of Toronto, Toronto, Canada; 7 BGI-Shenzhen, Beishan Industrial Zone, Yantian District, Shenzhen, 518083 China; 8 Department of Medicine and Department of Biological Sciences, The University of Alberta, Edmonton, AB T6G 2E1 Canada

**Keywords:** Computational biology and bioinformatics, Evolution

## Abstract

C_4_ photosynthesis is a remarkable complex trait, elucidations of the evolutionary trajectory of C_4_ photosynthesis from its ancestral C_3_ pathway can help us better understand the generic principles of the evolution of complex traits and guide the engineering of C_3_ crops for higher yields. Here, we used the genus *Flaveria* that contains C_3_, C_3_–C_4_, C_4_-like and C_4_ species as a system to study the evolution of C_4_ photosynthesis. We first mapped transcript abundance, protein sequence and morphological features onto the phylogenetic tree of the genus *Flaveria*, and calculated the evolutionary correlation of different features; we then predicted the relative changes of ancestral nodes of those features to illustrate the major events during the evolution of C_4_ photosynthesis. We found that gene expression and protein sequence showed consistent modification patterns in the phylogenetic tree. High correlation coefficients ranging from 0.46 to 0.9 among gene expression, protein sequence and morphology were observed. The greatest modification of those different features consistently occurred at the transition between C_3_-C_4_ species and C_4_-like species. Our results show highly coordinated changes in gene expression, protein sequence and morphological features, which support evolutionary major events during the evolution of C_4_ metabolism.

## Introduction

Elucidating the evolutionary and developmental processes of complex traits formation is a major focus of current biological and medical research. Most health-related issues, including obesity and diabetes, as well as agricultural challenges, such as flowering time control, crop yield improvements, and disease resistance, are related to complex traits^1–3^. Currently, genome-wide association studies are applied in the study of complex traits. Putative genes or molecular markers are then evaluated by a reverse genetics approach to identify those influence the complex traits. C_4_ photosynthesis is a complex trait that evolved from C_3_ photosynthesis. When compared with C_3_ plants, C_4_ plants have higher water, nitrogen and light use efficiencies^4^. Interestingly, C_4_ photosynthesis has evolved independently more than 66 times, representing a remarkable example of convergent evolution^5^. Accordingly, C_4_ evolution is an ideal system for the investigation of the mechanisms of convergent evolution of complex traits.

Change to C_4_ photosynthesis is associated with a number of biochemical, cellular and anatomical modifications when compared with the ancestral C_3_ photosynthesis^6,7^. In C_3_ photosynthesis, CO_2_ is fixed by ribulose-1,5-bisphosphate carboxylase/oxygenase (Rubisco), whereas in dual-cell C_4_ photosynthesis, CO_2_ is initially fixed into a four-carbon organic acid in mesophyll cells (MCs) by phospho*enol*pyruvate carboxylase (PEPC)^8^. The resulting four-carbon organic acid then diffuses into the bundle-sheath cells (BSCs)^9^, where CO_2_ is released and fixed by Rubisco. Hence, C_4_ photosynthesis requires additional enzymes in CO_2_ fixation in addition to those already functioning in C_3_ photosynthesis, including PEPC, NADP-dependent malic enzyme (NADP-ME), and pyruvate, orthophosphate dikinase (PPDK)^8^. In dual-cell C_4_ photosynthesis, CO_2_ is concentrated in BSCs that are surrounded by MCs, forming the so-called Kranz anatomy^10–12^. Compared with C_3_ leaf anatomy, Kranz anatomy requires a spatial rearrangement of MCs and BSCs, cell size adjustment for increased numbers of organelles, larger organelles and metabolite transfer between the two cell types, and a reduction in distance between leaf veins.

Much of the current knowledge regarding the evolution of C_4_ photosynthesis was gained through comparative studies in terms of physiology and anatomy by using genera that have both C_3_ and C_4_ species, as well as species performing intermediate types of photosynthesis^7,13,14^. Among these, the genus *Flaveria* has been promoted as a model for C_4_ evolution studies^15^, because the genus includes 23 known species which represent different photosynthetic types, including C_3_, C_4_ and different intermediate photosynthetic types^16,17^. *Flaveria* C_3_-C_4_ intermediate species are characterized by reduced photorespiration, lower CO_2_ compensation points compared to C_3_ species and partial to complete Kranz anatomy^15,17,18^. C_3_-C_4_ intermediate species are further divided into type I C_3_-C_4_ and type II C_3_-C_4_, the former reduces carbon loss solely relying on the photorespiratory CO_2_ concentration cycle as a result of reallocation of glycine decarboxylase in the BS tissues, whereas the later performs a partial C_4_ cycle as a result of increased enzyme activities of PEPC and NADP-malic enzyme^19,20^. C_4_-like intermediate species are featured by assimilating the majority of CO_2_ through the C_4_ cycle, but lacking a strict compartmentation of C_4_ enzymes between MC and BSC, and a small fraction of CO_2_ is initially fixed by Rubisco^20,21^.

The evolution of C_4_-related morphological, anatomical and physiological features has been well studied in this genus over the last 40 years^15,22–24^. The molecular evolution of several key C_4_ enzymes have been reported in this genus^25–27^, however, the molecular evolution of most C_4_ related genes is largely unknown. Besides, the evolutionary relationship between the C_4_ related genes and morphology features is not clear so far. In this study, we combined transcriptome data and published morphological data, together with the recent phylogenetic tree of the genus *Flaveria*^28^, to systematically investigate the key molecular events and evolutionary paths during the C_4_ evolution.

## Results

### Transcriptome assembly and quantification

RNA-Seq data of 31 samples of 16 *Flaveria* species were obtained from the public database Sequence Read Achieve (SRA) of the National Center for Biotechnology Information (NCBI) (Table S1). The 16 species represented two C_3_ species, seven C_3_-C_4_ intermediate species, three C_4_-like species and four C_4_ species^19,21^ (Table S1). On average, 42,132 contigs (from 30,968 to 48,969) were assembled with N50 ranging from 658 to 1208 bp among the 16 species (Table S2). The distribution of the contig length is similar in the 16 species with a peak at 360 bp (Fig. S1).

Since *Flaveria* is a eudicot genus, we used *Arabidopsis thaliana* (Arabidopsis) as a reference to annotate *Flaveria* transcripts. On average, 58.91% of *Flaveria* contigs had orthologous genes in Arabidopsis. Considering the large evolutionary divergence between Arabidopsis and *Flaveria*, which was estimated to be 120 million years (http://timetree.org/), we then estimated the accuracy of annotation by examining whether the transcripts annotated to be the same genes were from same orthologous groups. Specifically, we used OrthoFinder^29^ to predict the orthologous groups based on annotated genes of *Flaveria* species and then calculated the consistence between our gene annotation and orthologous groups. Specifically, for each orthologous group, we calculated the percentage of genes with the same annotation. For example*,* for the orthologous group of PPDK(AT4G15530), 29 transcripts from *Flaveria* and one gene (AT4G15530) with six transcripts from Arabidopsis were clustered in this orthologous group. All of the 29 transcripts from *Flaveria* were annotated as AT4G15530 (Fig. S2A). Therefore, the consistency of our annotation is 100% (29/29) for this orthologous group. Our result showed that 80% of the total 28,164 orthologous groups has a consistency higher than 90% (Fig. S2).

Transcript abundance was calculated as fragments per kilobase of transcript per million mapped reads (FPKM) (see Methods). The total transcriptome-level comparison revealed higher Pearson correlations in overall transcript abundance in leaf samples from the same species than those of different organs from the same species, regardless of sources (Fig. S3). Specifically, leaves from different developmental stages or from different labs are more closely correlated than leaf samples from different species, or than mean values of pair-wise correlations across all 27 leaf samples (T-test, P < 0.05) (Fig. S4). As a result, 13,081 Arabidopsis orthologs were detected in at least one of the 16 *Flaveria* species, and 12,215 were kept with the maximum FPKM in 16 species ≥ 1 FPKM.

### Investigate the possibility of samples used in this study being hybrid

**Figure 1:**
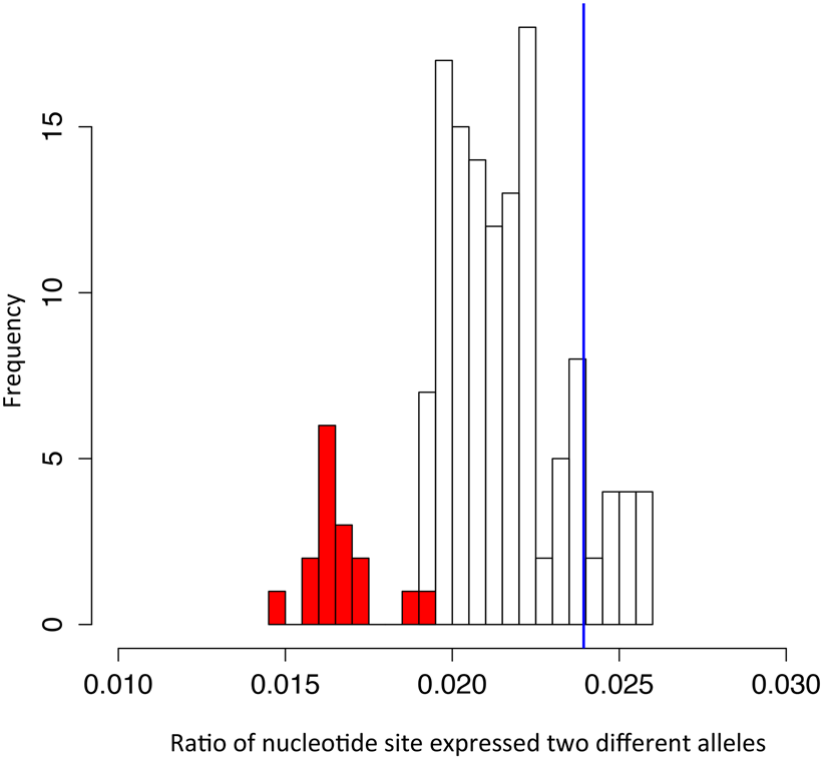
Estimation of the probability of RNA-Seq data from hybrid species. The bars show the distribution of ratio of nucleotide sites expressing two different alleles (mixed site). Mixed RNA-Seq samples are generated by pair-wise mixing RNA-Seq data of 16 *Flaveria* species, which mimics the case of hybridization. The ratio of mixed site in the mixed RNA-Seq samples is showed in grey bars (positive control). The ratio of mixed site of 16 *Flaveria* species is showed in red bars (real causes). The ratio of hybrid sample F. pringlei*, was represented in blue vertical line.

Considering that intermediacy of traits in the intermediate species may result from a possible hybridization between species with one parent being C_3_ and another parent being C_4_ or intermediate species^30^, we investigated whether the intermediate species used in this study are intermediate species or a hybrid offspring. The hybrid offspring is characterized as expressing different alleles at one DNA site; therefore, we calculated the percentage of DNA sites that expressed different alleles, which is termed as mixed site. The percentage of mixed site was then compared to the positive background generated by pair-wisely mixing of RNA-Seq data of 16 *Flaveria* species. Our results showed that the known hybrid sample F. pringlei* originated from *F. pringlei* × *F. angustiflolia* in^28^ showed significantly higher percentage of mixed site than background (Binomial test, P < 0.001), whereas, other species showed significantly lower percentages of mixed sites than background (Binomial test, P < 0.001) (Fig. 1). Thus, our data showed that species used in this study are not hybrids and can be used for evolutionary study.

**Figure 2:**
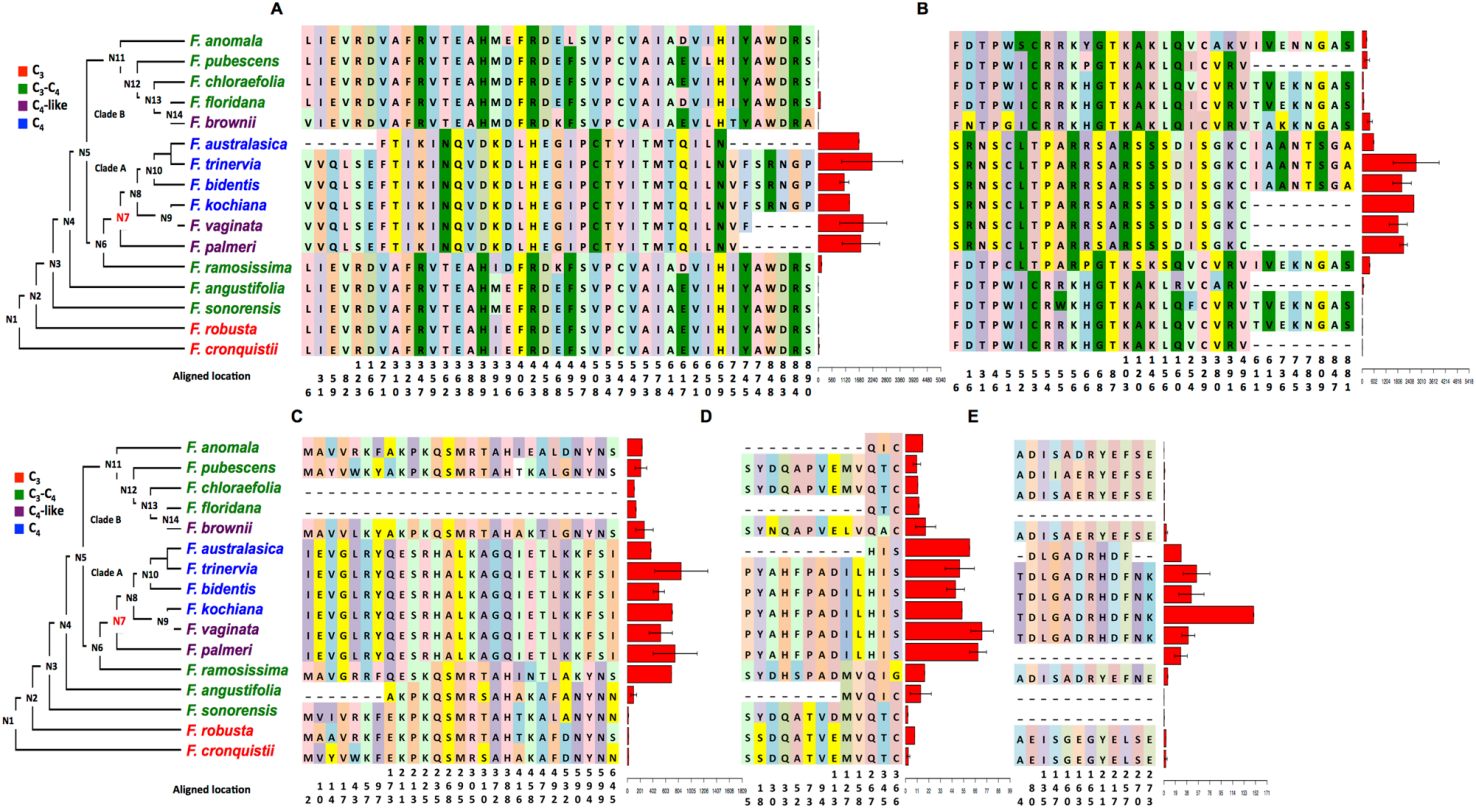
Modifications in genes in C_4_ pathway in predicted protein sequences and transcript abundances mapped to the *Flaveria* phylogeny. The predicted amino acid changes and the transcript abundance (FPKM) of the genes encoding the enzymes in C_4_ pathway are shown. Only the amino acid residues predicted to be different between C_3_ and C_4_ species are superimposed on the recent published phylogeny of *Flaveria*. The colors of amino acid residues have no meaning and are only for visualization purposes. Numbers below the amino acids indicate the location sites in the multiple sequence alignments. FPKM values with standard errors are shown to the right of the amino acid changes as red bars. (**A**) phospho*enol*pyruvate carboxylase (PEPC); (**B**) pyruvate orthophosphate dikinase (PPDK); (**C**) NADP-malic enzyme (NADP-ME); (**D**) pyruvate orthophosphate dikinase regulatory protein (PPDK-RP); (**E**) phospho*enol*pyruvate protein kinase A (PPCKA). Protein sequences from UniprotKP are: *F. trinervia* PEPC, P30694; *F. bidentis* PPDK, Q39735; *F. brownii* PPDK, Q39734; and *F. trinervia* PPDK, P22221. Sequence alignments are available in Additional file 2.

We used the phylogenetic tree of the genus *Flaveria*^28^ to illustrate the molecular evolution of C_4_ photosynthesis. We numbered each node of the phylogenetic tree in an order as showed in Fig. 2, usually, the more ancient a node is, the lower number it will be given. The number begins with N1 which refers to the common ancestor of all *Flaveria* species in the phylogenetic tree, N3 refers to the common ancestor on all intermediate and C_4_ species, at which intermediate species first evolved. N7 is the common ancestor of C_4_-like and C_4_ species in clade A, where a completed C_4_ cycle evolved. The nodes of clade B were numbered sequentially following clade A (Fig. 2).

### The modified genes: genes showed differences in gene expression and protein sequence between C_3_ species and C_4_ species

We first identified the modified genes, which were defined as genes show differences in both gene expression and protein sequence between C_3_ and C_4_ species. We first calculated the differentially expressed (DE) genes between C_3_ and C_4_ species in the way of comparing three C_3_ samples with 8 C_4_ samples (see Methods), which resulted in 896 DE genes (“BH” correlated *P* < 0.05) (Additional file 3). We next investigated transcriptome-wide amino acid changes predicted from orthologues of C_3_ and C_4_ *Flaveria* species using the process shown in Fig. S5. Briefly, an amino acid difference was classified as a change if the orthologous from the two C_3_ species (*F. robusta* and *F. cronquistii*) contained the same predicted amino acid at a position, but was different from the corresponding position in orthologs from at least two C_4_ species (among *F. kochiana*, *F. bidentis*, *F. trinervia* and *F. australasica*), the detailed process of identifying amino acid changes were described in Supplemental methods. As a result, we obtained 1,018 genes encoding at least one amino acid change between C_3_ and C_4_ *Flaveria* species. 56 out of these 1,018 genes also showed significantly differentially expression between C_3_ and C_4_ species, which was termed as modified genes.

### The modified genes showed coordination in major changes in the C_4_ evolutionary pathway in the genus *Flaveria*

In addition to C_4_ pathway, cyclic electron transport chain (CET) and photorespiratory pathway are reported to be related to the evolution of C_4_ photosynthesis. We manually selected genes from these three pathways from the literatures^23,24,31^ and then tested whether genes from these pathways were significantly enriched in the 56 modified genes (Table S4). Results showed that genes related to C_4_ photosynthesis pathway and genes related to CET were significantly enriched in the 56 modified genes. (“BH” correlated *P* < 0.05, *Fisher’s* exact test, Supplemental methods). We systematically discussed these genes and their changes during C_4_ evolution in *Flaveria* with gene expression and predicted protein sequences.

#### Genes encoding proteins associated with the C_4_ pathway

Nine genes encoding proteins associated with the C_4_ pathway were identified, including those encoding three C_4_ cycle enzymes, PEPC, PPDK and NADP-ME, two regulatory proteins, PPDK regulatory protein (PPDK-RP) and PEPC protein kinase A (PPCKA), two aminotransferases, Alanine aminotransferase (AlaAT) and aspartate aminotransferase 5 (AspAT5), and two transporters, BASS2 and sodium: hydrogen (Na^+^/H^+^) antiporter 1 (NHD1) (Table 1). In terms of protein sequence, on average, 66.0% (from 33.33% to 86.7%) of amino acid changes in C_4_ species occurred at N7 for all of the nine genes (Fig. 2, Figs. S6, Table 1). For example, PEPC in the C_4_ *Flaveria* species had 41 predicted amino acid changes compared with those in the C_3_ species, which were mapped onto the *Flaveria* phylogeny determined by Lyu et al.^28^. One of the predicted changes occurred at N6 (D396 in C_4_ species, hereafter D396), and 34 occurred at N7 (Fig. 2A). The six other predicted amino acid changes occurred at N7 or after N7, although the incomplete assembly of PEPC transcripts from *F. palmeri* and *F. vaginata* did not allow resolution of the predicted amino acid sequences. These results suggest a major evolutionary event in the protein sequence at N7 for C_4_ enzymes.

**Table 1:** Proteins showing differences in amino acid sequence between C_3_ and C_4_ *Flaveria* species and the relative changes in their cognate transcripts.

Ortholog in *A. thaliana*	Genes encoding proteins involved in	Mean FPKM (C4 )/mean FPKM (C3 )	FDR (EdgeR)	Length in Fcro (Frob)^α^	Protein length in *A. thaliana* (aa)	aa changes						Stage of key change(s) in sequence	Stage of key change(s) in FPKM^β^
						Total aa change(s)	Before N 5	At N5	At N6	At N7	After N7		
**Gene in C4 pathway**													
AT3G14940	PEPC	85.58	2.78E−06	966	968	41			1	>=34		N7	N7
AT4G15530	PPDK	123.6	9.10E−09	958	963	31			2 + 6-aa REP	>=15		N7	N7
AT1G79750	NADP-ME	26.64	6.64E−08	647	646	27		1	8	18		N7	N3 and N6
AT4G21210	PPDK-RP	7.57	1.63E−03	402	403	13	1		4	7		N7	N7
AT3G04530	PEPC-k	88.78	2.93E−03	281	278	12	3		2	7		N7	N7
AT1G72330	AlaAT	9.63	1.57E−04	544	553	9			2	7		N7	N3 an N6
AT4G31990	AspAT5	36.67	4.34E−06	459	453	3			1	1	1	N7	N3 and N7
AT2G26900	BASS2	39.12	5.30E−07	415	409	14			2	12		N7	N7
AT3G19490	NHD1	51.19	8.49E−07	576	576	15			2	13		N7	N7
**Gene related to electron transport chain**													
AT4G22890	PGR5-like	7.1	4.70E−02	328	324	10+17-aa INS	1		2+17-aa INS	7		N6	N7
AT1G14150	NdhL2/PnsL2	3.71	2.13E−02	190	190	4	2		1	1		before N5	N7
AT2G04039	NdhV	9.17	1.73E−02	227	199	8		1	6	1		N6	N3
AT5G43750	Ndh18/PnsB5	6.8	6.27E−02	224	212	3			2	1		N6	N8
AT5G21430	NdhU/CRRL	8.55	3.11E−03	215	218	4			4			N6	N7
AT4G37925	NdhM	7.01	5.24E−02	209	217	3			1	2		N7	N8
AT1G15980	Ndh48/PnsB1	8.6	1.77E−02	465	461	7	1		4	2		N6	N7
AT1G18730	NdhB4/PnsB4	8.8	9.57E−03	182	174	5		1	2	2		N6 and N7	N7
AT5G52100	CRR1	4.05	6.78E−03	302	298	5			1	1	3	after N7	N3
**Gene in photorespiration pathway**													
AT5G35630	GSL1	0.08	2.15E−02	430	430	8	3		1	2	1	N7	N7
AT1G32470	GDC-H	0.23	*7.29E−01*	162	166	6+2-aa INS + 1-aa INS				5 + 2-aa INS + 1-aa INS	1	N7	N7
AT4G37930	SHM	0.16	*4.08E−01*	517	517	8	3			5	1	N7	N7
AT1G80380	GLYK	0.49	*3.61E−01*	443	456	8			2	6	1	N7	N7
AT5G04140	GOGAT	0.57	*1.32E−01*	1616	1648	18	4		>=1	>=5	2	N7	N7

In terms of gene expression, all nine genes showed higher transcript abundance in C_4_ species than in C_3_ species and a comparable level in C_4_-like and C_4_ species (Table 1). To calculate the relative gene expression changes of each ancestral node, the FPKM values of each ancestral node were predicted and the relative difference were calculated (see Methods). In general, C_4_ species showed a 7.6-fold to 123.6-fold of FPKM values compared with C_3_ species. Similar to the pattern of changes of protein sequences, seven of the nine genes showed that the biggest relative changes in gene expression occurred at N7, whereas, both NADP-ME and AlaAT showed the biggest relative changes at two nodes of N3 and N6 with comparable levels (Fig. 2C and Fig. S6A). Our results therefore suggest that the genes encoding proteins associated with C_4_ pathway showed highly coordinated modification patterns in protein sequence and gene expression at N3, N6 and N7 during the evolutionary change to C_4_ photosynthesis, while the majority of the predicted amino acid changes occurs at the N7.

#### Genes encoding proteins involved in CET chain

**Figure 3:**
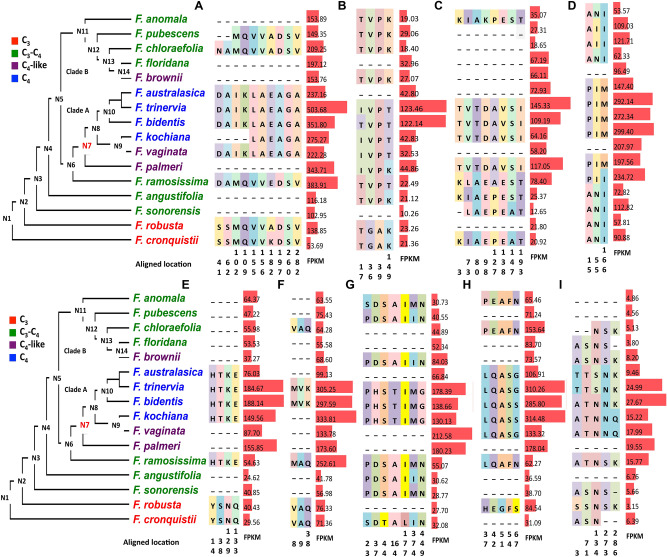
Modifications in the predicted amino acid sequences of proteins involved in cyclic electron transport and transcript abundances of the cognate transcripts mapped to the *Flaveria* phylogeny. Changes in predicted amino acid sequence in proteins involved in cyclic electron transport chain and abundances (FPKM) of their cognate transcripts in C_4_ and C_3_ *Flaveria* species are shown. Only the amino acid residues predicted to be different between C_3_ and C_4_ species are superimposed recent published phylogeny of *Flaveria*. The marked colors of amino acid residues have no meaning and are only for visualization purposes. Numbers below the amino acids indicate the location site in the multiple sequence alignments. FPKM values with standard errors are represented to the right of the amino acid changes as red bars. (**A**) Protein gradient regulation 5 like protein (PGR5-like); (**B**) NADH dehydrogenase-like (Ndh) L2 subunit (Ndh L2); (**C**) NdhV; (**D**) Ndh16; (**E**) NdhU; (**F**) NdhM; (**G**) Ndh48; (**H**) NdhB4; (**I**) chlororespiration reduction 1 (CRR1). The sequence alignments are available in Additional file 2.

We identified genes encoding nine proteins that function in the CET chain, namely, proton gradient regulation 5 like (PGR5-like), the chloroplast NAD(P)H dehydrogenase complex (Ndh) L2-2 (NdhL2-2), NdhV, Ndh18, NdhU, NdhM, Ndh48, NdhB4, and chlororespiratory reduction 1 (CRR1). The transcripts encoding all the nine proteins showed higher abundances in C_4_ species than C_3_ species (Fig. 3 and Table 1). Compared to the genes encoded protein in the C_4_ pathway, the genes encoding proteins involved in CET chain showed the biggest changes at diverse nodes rather than at a single node. Specifically, the major changes of predicted protein sequences occurred at N6 and N7 and that of FPKM occurred at N3, N7 and N8 (Table1). Besides this, the modification of protein sequence and FPKM appears to be less coordinated in genes involved in CET chain, for example, the major change of PGR5-like occurred at N6 in predicted protein sequence and at N7 in FPKM (Fig. 3A).

#### Genes encoding proteins in the photorespiratory pathway

The establishment of the photorespiratory pump (C_2_ photosynthesis) is reported to be a prerequisite for the evolution of C_4_ photosynthesis based on theoretical modeling^32^. Therefore, we also investigate genes involved in the photorespiratory pathway in terms of predicted protein sequence and FPKM.

**Figure 4:**
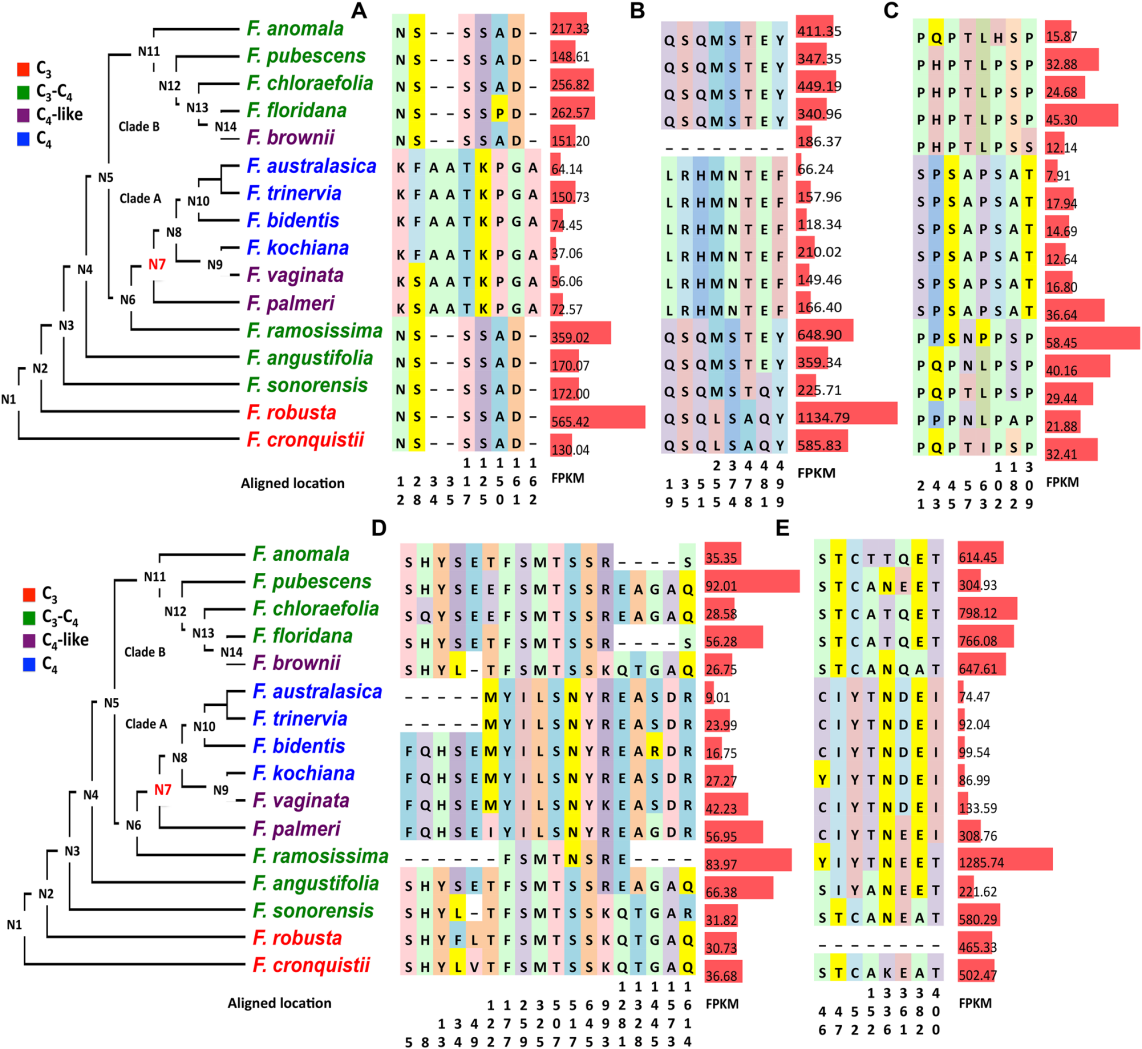
Modifications in photorespiratory protein predicted amino acid sequences and cognate transcript abundances mapped to the *Flaveria* phylogeny. The predicted amino acid changes in photorespiratory proteins between C_4_ and C_3_ *Flaveria* species and the transcript abundance (FPKM) of genes encoding the proteins are shown. Only the amino acid residues that are predicted to be different between C_3_ and C_4_ species are superimposed on the recent published phylogeny of *Flaveria*. The marked colors of amino acid residues have no meaning and are only for visualization purposes. Numbers below the amino acids indicate the location site in the multiple sequence alignments. FPKM values with standard errors are represented to the right of the amino acid changes as red bars. (**A**) Glutamine synthetase like 1 (GSL1); (**B**) glycine decarboxylase complex H subunit (GDC-H); (**C**) serine hydroxymethyltransferase (SHM); (**D**) glycerate kinase (GLYK); (**E**) glutamine synthetase and glutamine oxoglutarate aminotransferase (GOGAT).

One protein involved in photorespiration was included in the 56 modified genes, namely, glutamine synthetase-like 1 (GSL1). Moreover, four other proteins in this pathway showed abundant amino acid changes between C_3_ and C_4_ species, namely, glycine decarboxylase complex (GDC) H subunit (GDC-H), serine hydroxymethyltransferase (SHM), glycerate kinase (GLYK), glutamine synthetase and glutamine oxoglutarate aminotransferase (GOGAT) (Fig. 3, Table 1). In general, the predicted amino acid substitution patterns of these five proteins were similar to those observed in the above-described proteins in C_4_ pathways, with the major predicted amino acid changes in C_4_ species occurring at N7 (Fig. 4, Table 1), *e.g.,* 16 of 18 in GOGAT occurred at N7 (Fig. 4D). Generally, proteins in the photorespiratory pathway showed fewer predicted amino acid changes than those in the C_4_ pathway.

The abundance of transcripts encoding these five photorespiratory enzymes was comparable to those in C_3_ and C_3_-C_4_ species, and higher than that in C_4_ species (Fig. 4A–E). When compared with genes encoding C_4_ pathway proteins, those encoding photorespiratory proteins showed larger differences between C_4_-like and C_4_ species in clade A in terms of gene transcript abundance and protein sequence. The greatest reduction of FPKM in these five genes was observed at N7 (Fig. 4, Table 1). Thus, this suggested that the genes encoded proteins associated with the photorespiratory pathway also showed coordinated changes in protein sequence and gene expression during the evolution of C_4_ photosynthesis, and with the largest number of changes occurring at N7.

### Physiological and anatomical characteristics related to C_4_ photosynthesis show coordinated changes along the C_4_ evolutionary pathway in *Flaveria*

**Figure 5:**
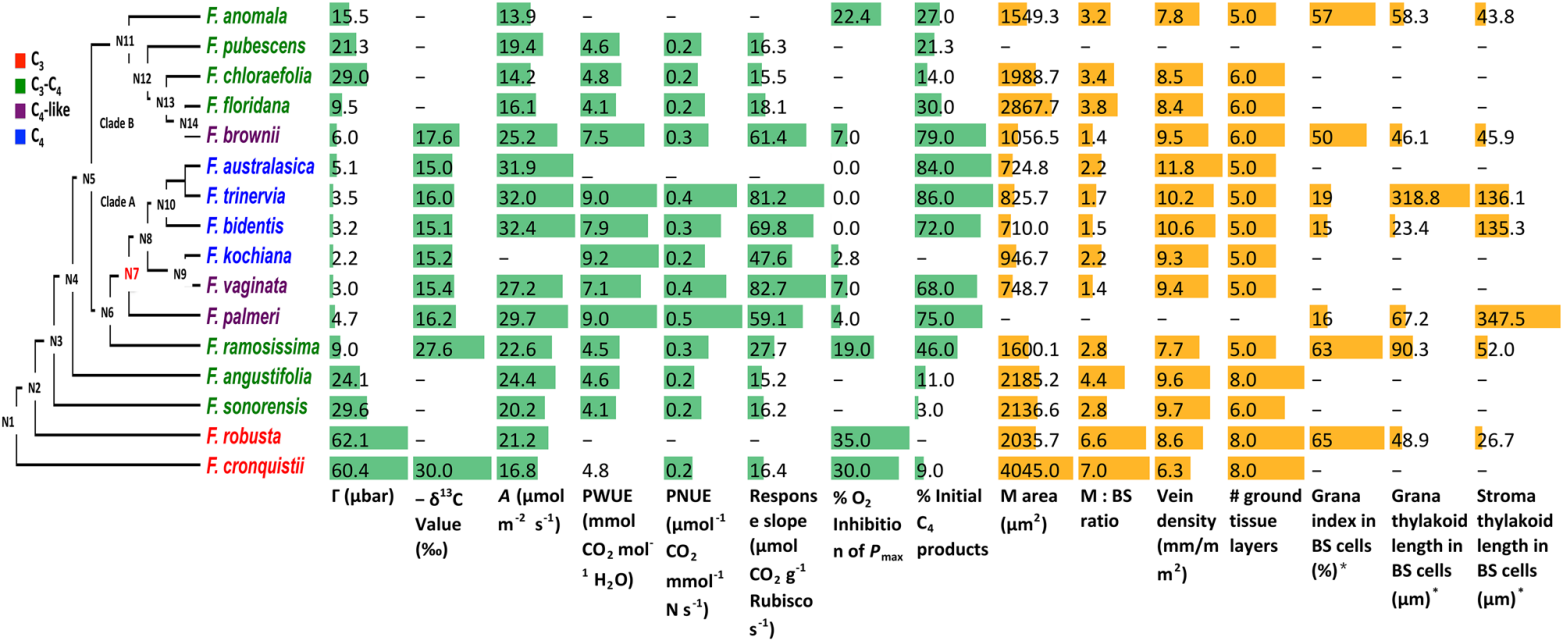
Changes in physiological and anatomical traits mapped onto the *Flaveria* phylogeny. Overall, C_4_-related physiological (green bars) and anatomical traits (orange bars) showed a step-wise change along the *Flaveria* phylogenetic tree; however, a number of the traits showed greater more significant changes at certain nodes. *Grana index: total length of grana/total length of thylakoid membrane X 100. (*Γ* CO_2_ compensation point, *A* CO_2_ assimilation rate, *PWUE* instantaneous photosynthetic water use efficiency, *PNUE* instantaneous photosynthetic nitrogen use efficiency, *response slope* slope of the response of net CO_2_ assimilation rate versus leaf Rubisco content, *M* mesophyll, *BS* bundle sheath.) Data are from references as given in the Methods.

To investigate whether C_4_ related physiological characteristics also underwent coordinated changes during the evolution of C_4_ photosynthesis in *Flaveria*, physiological characteristics taken from the literature^18,21,33^ were mapped onto the *Flaveria* phylogeny (Fig. 5). The results revealed a step-wise change for most of the characteristics along the phylogenetic tree as previously suggested^15,18,21,33^ (Fig. 5). However, coordinated and abrupt changes were observed for a number of features. A major change in CO_2_ compensation point (Γ) in *Flaveria* was first seen at N3, where the most ancestral C_3_-C_4_ species, *F. sonorensis* emerging showing a decrease in Γ from 62.1 μbar of its closest C_3_ relative *F. robusta* to 29.6 μbar (Fig. 5). The greatest changes in Γ in clade A occurred at N6, which showed a decrease in Γ from 24.1 μbar in *F. angustifolia* (C_3_-C_4_) to 9.0 μbar in *F. ramosissima* (C_3_-C_4_), followed by N7, where a decrease in Γ from 9.0 μbar in *F. ramosissima* (C_3_-C_4_) to 4.7 μbar in *F. palmeri* (C_4_-like) was observed. The greatest decrease of Γ in clade B was observed between the two C_3_-C_4_ species, *F. floridana* and *F. chloraefolia* (C_3_-C_4_), where there was a decrease from 29 μbar to 9.5 μbar (Fig. 5). For photosynthetic water use efficiency (PWUE), photosynthetic nitrogen use efficiency (PNUE) and the slope of the response of the net CO_2_ assimilation rate (*A*) versus Rubisco, the biggest changes occurred at N7 with increases of around twofold. In contrast, the percentage of ^14^C fixed into four carbon acids showed no clear trend across the phylogenetic tree, although 3.91-fold and 1.76-fold increases were seen at N6 and N7, respectively. Interestingly, changes in all of these traits uniformly occurred at *F. brownii* in clade B, the only C_4_-like species within this clade. Consequently, those data suggest that although there were gradual changes in physiological features along the C_3_, C_3_-C_4_, C_4_-like and C_4_ trajectory, there are apparent major modifications at N3, N6 and N7 in these physiological traits across the *Flaveria* phylogeny (Fig. 5).

Anatomical traits^15,34^ were mapped onto the *Flaveria* phylogeny to investigate how these features were modified along the evolution of C_4_ (Fig. 5). For both the area of MCs and the ratio of the area of MCs to that of BSCs (M: BS), the greatest modifications across the phylogeny were found between *F. brownii* (C_4_-like) and *F. floridana* (C_3_-C_4_), with a similar degree of change for both characteristics (2.7-fold, Fig. 5). Anatomical data for *F. palmeri* (C_4_-like) in clade A are not available, however, large differences in anatomical features were found between the C_4_-like *F. vaginata* and C_3_-C_4_ *F. ramosissima*^15^. The modification of MC area first occurred at N2 which showed a 1.9-fold difference between *F. robusta and F. cronquistii* followed by a 2.1-fold of difference between *F. ramosissima* and *F. vaginata*. A major modification of the ratio of MC and BSC occurred at N2 with a 2.4-fold difference and followed by N6 with a 1.6-fold difference and N7 with a twofold difference. Therefore, similar to the evolutionary pattern of physiological features, large changes in anatomical features also emerged at N3, N6 and the transition between C_3_-C_4_ species and C_4_-like species. Interestingly, the ultrastructure of BSCs chloroplasts showed an abrupt change at N7, with a dramatic decrease in grana thylakoid length and an increase in stroma thylakoid length, whereas these features were comparable in the species at the base of tree and in clade B^34^.

### Coordinated changes of protein sequence, gene expression and morphology with a major evolutionary event at the transition between C_3_-C_4_ and C_4_-like species during the evolution of C_4_ species

**Figure 6:**
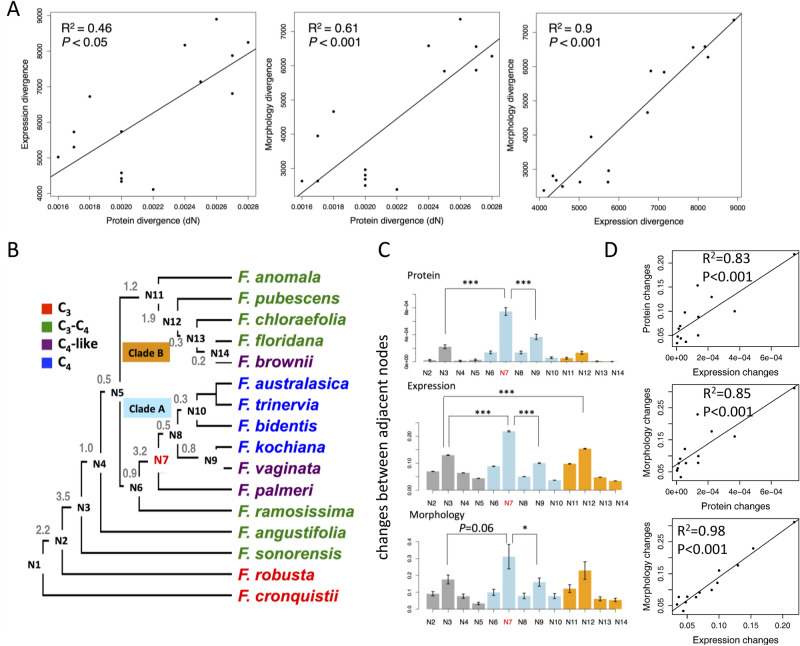
Coordinated evolution of protein sequence, gene expression and morphological traits with apparent major changes. Significant linear correlation between protein divergence, gene expression divergence and morphology divergence were showed in (**A**). Protein divergence was calculated as non-synonymous mutation (dN). Expression divergence and morphology divergence were calculated as Euclidean distance based on quantile normalized FPKM values calculated here and coded morphology values from Mckown et al*.* ^16^, respectively. All the relative divergences were the divergence between *F. cronquistii* and other *Flaveria* species. (**B**) The schema of *Flaveria* phylogenetic tree modified from Lyu, et al. ^28^, branch length between ancestral nodes were labeled in grey. (**C**) The relative changes of each ancestral node compared with its earlier ancestral node in protein sequence, gene expression and morphological traits. *P* values are from One-way ANOVA analysis followed by Tukey’s Post Hoc test and adjusted by *Benjamin-Hochberg* correction. The significant levels are: *: *P* < 0.05; **: *P* < 0.01; ***: *P* < 0.001. The bar colors in grey/blue/orange represent species from basal/clade A/clade B of phylogenetic tree, respectively. (**D**) Pearson correlations between changes of ancestral nodes in protein sequence, gene expression and morphological traits.

Our above analysis showed that C_4_ related genes and morphological features showed coordinated changes with an obvious major change at N7. Next, we asked whether species evolution also showed evolutionary coordination and major event during the evolution of species in protein sequence, gene expression and morphology. To answer this question, we calculated the divergence matrices for protein sequence, gene expression, and morphological features between *F. cronquistii* (at the most basal position on the *Flaveria* phylogenetic tree) and other *Flaveria* species. The protein divergence was calculated as the rate of non-synonymous substitutions (dN) of all the genes that were used to construct the *Flaveria* phylogenetic tree from^28^, the expression divergence was calculated as Euclidean distance of total expressed genes (see Methods), and the morphology divergence was calculated as Euclidean distance using previously coded morphology value from^16^, which includes 30 types of morphological traits, such as life history, leaf shape, head types. Our result showed a high linear correlation between the protein divergence, gene expression divergence and morphology divergence, in particular between gene expression divergence and morphology divergence (R^2^ = 0.9) (Fig. 6A). Thus, our results suggest a coordinated evolution of protein sequence, gene expression and morphology during species evolution. The linear correlation of gene expression divergence *vs* morphology divergence and protein divergence *vs* morphology divergence reflects that both gene expression changes and protein sequence changes are related to morphological changes during evolution^35,36^. Moreover, gene expression changes may be more directly related to morphological changes than protein sequence changes^37^. It is likely that changes of developmental programs might be mainly due to changes in gene expression levels while changes in the protein sequences might contribute more to changes in metabolism.

Next, we predicted the protein sequence, transcript abundance and coded morphology value of ancestral nodes, which were then used to calculate the relative change of the three parameters at each node (see Methods). Surprisingly, protein sequence and gene expression showed significantly more changes from N6 to N7 than changes during transitions between other nodes (*P* < 0.001, Tukey’s test, “BH” adjusted, the same as following), and the morphology showed the most changes from N6 to N7 with a marginal significant *P* value (*P* = 0.06) (Fig. 6B,C). We found high correlations between divergences in protein sequence, gene expression and morphological traits (Fig. 6D), implying that evolutionary coordination of major events on whole transcriptomic level also occurred in species evolution.

We then asked whether the major events were results of a long evolutionary time. We found positive correlations between the divergence (in protein sequence, gene expression and morphology) and branch length. For protein sequence, gene expression and morphological traits, their Pearson correlation coefficients with branch length are 0.35, 0.66 and 0.59, respectively (Fig. S7A). We then normalized the divergence between nodes to branch length to calculate change rate by assuming modification of protein sequence, gene expression and morphology have a linear relationship with evolutionary time. Results showed that N7 does not show the highest change rate, whereas, younger nodes tend to have higher change rates (Fig. S7B). For example, N14 showed higher modification rates than other nodes in gene expression and morphology. This is reasonable, because recovery mutations occurred during evolution, and modification rate was likely to be underestimated within a long evolutionary time. Besides, the relationship between amino acid substitution and evolutionary time may be non-linear but follow complex models^38^. Consistently, we found that change rates of protein sequence are neither correlated with that of gene expression nor with that of morphological traits. Whereas, change rates of gene expression are positively correlated with that of morphological traits (R2 = 0.91, P value < 0.001) (Fig. S7C). The large number of changes between N6 and N7 are likely a result of a long evolutionary time.

## Discussion

### Evolutionary coordination of different features towards a functional C_4_ metabolism

Compared to C_3_ photosynthesis, the evolution of C_4_ photosynthesis resulted in the acquisition of many new features in gene expression, protein sequence, morphology and physiology (Figs. 2, 3, 4, 5)^39^. Coordinated changes on these features were required at key transitions. This is because although C_4_ photosynthesis can gain higher photosynthetic energy conversion efficiency, highly specialized leaf and cellular anatomical features and biochemical properties of the involved enzymes are required. For example, increased cell wall thickness at the bundle sheath cell and decreased sensitivity of PEPC to malate inhibition are needed for C_4_ plants to gain higher photosynthetic rates^40,41^. Furthermore, to gain higher photosynthetic efficiency in C_4_ plants, the ratio of the quantities of Rubisco content in BSCs and MCs is also critical^42^. In theory, if the C_4_ decarboxylation evolves before all of the other accompanying changes required or C_4_ photosynthesis, leaves will experience high leakage, *i.e.*, costing ATP for a futile cycle without benefit to CO_2_ fixation. This will inevitably lead to lower quantum yield and a potential driving force for purifying selection. Further evidence for possible purifying selection comes from the observation that genes with cell-specific expression, such as PEPC, PPDK, and NADP-ME, displayed more changes in their predicted protein sequences than ubiquitously expressed genes, such as NDH components (Table 1, Additional file 3). This is because, as discussed earlier, the redox environments between BSCs and MCs might have changed dramatically during the evolution of the C_4_ cycle, with one of the most likely changes being a more acidic environment due to increased production of Oxaloacetic acid (OAA) and malate. Under such conditions, it is required for enzymes to alter their amino acid sequences to adapt to the new cellular environments. Concurrent changes of gene expression and protein sequence have also been demonstrated previously in animals^36,43^.

The identified changes in the protein sequences, including amino acid changes, insertions, and deletions (Table 1, Figs. 2, 3, 4), may enable the enzymes or proteins to improve biochemical and regulatory properties to meet the demands of an altered cellular environment, for example, the increased fluxes through the C_4_ cycle^44^. It is worth mentioning that some of these predicted amino acid changes have been reported to be functional, such as that the S774 and G884 residues in C_4_ PEPC determines the high substrate affinity and low inhibitor affinity of this enzyme, respectively^25,45^. Besides this, many of the predicted amino acid changes are in residues that can be post-translationally modified, for example, six residues in PPDK changed to Serine (S) in C_4_ species, which can all be target for phosphorylation and hence functional modification.

### Major evolutionary events along the C_4_ evolution in the *Flaveria* genus

Among the ancestral nodes leading to the C_4_ emergence in clade A, N7, which is the most recent common ancestral node of C_4_-like and C_4_ species in clade A, shows the biggest change in protein sequence, gene expression and morphology in both C_4_ specific features and also non-C_4_ specific features (Table 1, Figs. 2, 3, 4, 5, 6). There were also apparent changes in these features at N3 and N6. These three nodes reflect three critical stages in the emergence of C_4_ metabolism. Firstly, at N3, *i.e.,* during the emergence of C_3_-C_4_ species, there were many changes in gene expression, protein sequence and morphology. One of the most important events during this phase is the re-location of GDC from MSCs to BSCs based on earlier western blot data^13,46^. Here we found that SHM showed decreased expression while most of other photorespiratory related enzymes showed little changes (Fig. 4). Similarly, at this step, the majority of the C_4_ related genes showed little changes (Fig. 2). However, modification of gene expression levels and protein sequences on transcriptome level and no-C_4_ morphology features suggests that there are a large number of changes at N3 (Fig. 6), and there is also greater decrease of CO_2_ compensation point at this stage (Fig. 5).

C_3_-C_4_ species were also reported in several other genera from both monocotyledonous and dicotyledonous plants, such as the monocotyledonous *Alloteropsis*^47^, *Homolepis, Neurachne* and the dicotyledonous *Steinchisma*^48^ from monocot; *Moricandia*^49^, *Heliotropium*^50^ and *Mollugo*^51^. These C_3_-C_4_ species usually have a reduced CO_2_ compensation point, enlarged BSC, and their GDC-P subunit is predominately expressed in BSC. C_3_-C_4_ species in *Moricandia* do not show enhanced C_4_ cycle, besides, ^14^C labeling patterns for photosynthesis related metabolites are comparable to those in C_3_ species^49^, which may be equivalent to *Flaveria* C_3_-C_4_ species derived from N3.

N6, *i.e.*, during the emergence of type II C_3_-C_4_ *F. ramosissima* and C_4_-like species. is the stage where we found the third largest number of changes in C_4_ related features had occurred. At this stage, we observed large increase in transcript abundance in C_4_ genes (Fig. 2 & Fig. S5) and photorespiratory genes (Fig. 4), and that a dramatic increase in the percentage of ^14^C incorporated into the four carbon acids occurred (Fig. 5). The modification of photorespiratory genes might be related to the optimization of C_2_ cycle to decrease CO_2_ concentrating point, which can increase fitness of plants under conditions favoring photorespiration^52^. The concurrent modifications of C_4_ enzymes, such as PEPC, NADP-ME, PEPCKA, amongst others, which are also involved in nitrogen rebalancing, is consistent with the notation that C_4_ cycle might be evolved as a result of rebalancing nitrogen metabolism after GDC moving from MC to BSC^24^. The fact that there is little change in the δ^13^C in the C_3_-C_4_ intermediate as compared to that of C_3_ species suggests that the contribution of CO_2_ fixation following evolution of the C_4_ pathway is relatively minor, *i.e.*, less than 15%, estimated based on an δ^13^C value of -27.6 in *F. ramosissima* (Fig. 5), again supporting that the initial role of increased C_4_ enzymes is not for enhancing CO_2_ fixation. It is worth pointing out here that the measured initial carbon fixation in the form of C_4_ compound was 46% (Fig. 5); higher than those estimated based on the δ^13^C value. This is possibly because although malate releases CO_2_ into BSCs as a result of the nitrogen rebalancing pathway, most of the CO_2_ was not fixed by Rubisco, either due to the lack of sufficient Rubisco activity in BSCs or due to a lack of required low BSCs cell wall permeability to maintaining high CO_2_ concentration in BSCs.

N7, *i.e.*, during the emergence of C_4_-like and C_4_ species, witnesses abrupt changes for both the gene expression and proteins sequence and morphology (Figs. 2, 3, 4, 6). The majority of the C_4_ related genes showed the most modification in gene expression and protein sequence at N7, especially for genes in C_4_ cycle and photorespiratory pathway. Moreover, N7, at which C_4_-like species (clade A) appear, represents a dramatic shift of CO_2_ fixation from being dominated by a C_2_ concentrating mechanism to being dominated by a C_4_ concentrating mechanism. Based on the δ^13^C value in *F. palmeri*, the fixation through the C_4_ concentrating mechanism is up to 93%, which is consistent with the measured proportion of initial carbon fixation in the form of C_4_ compound (Fig. 5), suggesting at this step, the released CO_2_ in the BSCs can be largely fixed by Rubisco, whereas, the transition between C_4_-like to C_4_ process is an evolutionarily "down-hill" process and most optimization occurred through fine-tuning gene expression.

Genes from C_4_ pathway and photorespiration displayed the major evolutionary changes at N7 both in gene expression and protein level (Table 1). At the same time, we found morphological features, protein sequence and gene expression, which were not necessarily related to C_4_ photosynthesis also showed major changes at that stage (Fig. 6). This coincidence suggests that the C_4_ and photorespiratory pathway may be a main driving force in the evolution in *Flaveria*.

The genus *Flaveria* has been used as a model system to study the evolution and regulation of C_4_ photosynthesis^15–17^. Over the past 40 years, many labs conducted studies related to metabolism, physiology, anatomy, morphology, transcriptome and transcript regulation for different groups of *Flaveria* species^15,22–24^. Here, we combined data from different aspects for 16 *Flaveria* species with a purpose to examine whether the different C_4_ related traits evolve in a coordinated manner and at the same time to study the relationship between species evolution and C_4_ photosynthesis evolution. Here we caution that *Flaveria* plants from different labs were grown under different conditions, data from different labs may be not in complete accord. For examples, the RNA-seq data used here were originally generated from two labs (Supplemental methods), plants were grown under different conditions and RNA-seq data were sequenced from different strategies. Hence, we performed additional normalization to the FPKM to make gene expression comparable among different samples. For DE genes between C_3_ and C_4_ species, we applied calcNormFactors function embed in edgeR^53^ to calculate scaling factors and convert raw library sizes into effective library sizes. DE genes called here may be not exactly consistent with that called based on RNA-seq data from each one of the labs, but should reflect the major DE genes between C_3_ and C_4_ species.

## Materials and methods

### Data retrieval

RNA-Seq data of *Flaveria* species were downloaded from the Sequence Read Achieve (SRA) of the National Center for Biotechnology Information (NCBI). The source of RNA-seq data and plant grown conditions are detailed in Supplementary Methods. All accession numbers for RNA-Seq data are shown in Table S1.

CO_2_ compensation points (Γ) (except for *F. kochiana*), δ^13^C (except for *F.kochiana*), %O_2_ inhibition of P_max_ (except *F. kochiana*), and CO_2_ assimilation rates were from^21^. Γ, δ^13^ and %O_2_ inhibition of *F. kochiana* were from^33^. Data for % initial C_4_ products in total fixed carbon were from^54^. Data for PWUE, PNUE, and net CO_2_ assimilation rate (*A*) versus Rubisco content were from^33^. Data for M area, M:BS ratio, vein density and number of ground tissue layers were from^15^. The values of M area, M:BS ratio and vein density were measured from figures in McKown and Dengler^15^ with GetData (http://www.getdata-graph-digitizer.com). Mean values from 20 measurements were used. Ultrastructural data of BS cell chloroplasts were from Nakamura et al.^34^.

### Transcriptome assembly and quantification

Transcripts of *Flaveria* species generated with Illumina sequencing were assembled using Trinity (version 2.02)^55^ with default parameters (Table S1). Contigs of four *Flaveria* species from 454 sequencing data were assembled using CAP3^56^ with default parameters. In all cases, only contigs of at least 300 bp in length were saved. Transcript abundances of 31 *Flaveria* samples were analyzed by mapping Illumina short reads to assembled contigs of corresponding species and then normalized to the fragment per kilobase of transcript per million mapped reads (FPKM) using the RSEM package (version 1.2.10)^57^. Functional annotations of *Flaveria* transcripts were determined by searching for the best hit in the coding sequence (CDS) dataset of *Arabidopsis thaliana* (Arabidopsis) in TAIR 10 (http://www.arabidopsis.org) by using BLAST in protein space with E-value threshold 0.001. If multiple contigs shared the same best hit in CDS reference of Arabidopsis, then the sum FPKM of those contigs was assigned to the FPKM value of the gene in *Flaveria*.

To estimate the consistence of *Flaveria* gene annotation, we used OrthoFinder^29^ to predict the orthologous group based on the annotated *Flaveria* gene together with gene of Arabidopsis from TAIR10, and then calculated the consistence between gene annotation and orthologous group in two ways. (1) If the orthologous group contains Arabidopsis gene(s), the consistency was calculated as the percentage of genes that have the same annotation with the Arabidopsis gene(s). (2) If there the orthologous group does not contain Arabidopsis gene, we calculated the percentage of genes for each gene ID in this group, and the highest percentage was assigned to the consistency.

To make the FPKM comparable across different samples, we normalized the FPKM value by a scaling strategy as used by Brawand et al.^35^. Specifically, among the transcripts with FPKM values ranking in 20–80% region in each sample, we identified the 1000 genes that had the most-conserved ranks among 29 leaf samples, which were then used as an internal reference, and the transcript of each sample was normalized according to the mean value of these 1000 genes in the sample. We then multiplied all the FPKM values in all samples by the mean value of 1000 genes in the 29 leaf samples. The three samples from C_3_ species and eight samples from C_4_ species (Table S1) were used to recall differentially expressed (DE) genes applying edgeR^53^, and the Benjamini-Hochberg (“BH”) procedure was used in multiple testing correction with a threshold of *P* (“BH” corrected) to be 0.05.

### Investigation the species used in this study being from hybrid of two species

To investigate whether the intermediate species used in this study are from hybrid offspring of two species, DNA sites that expressed different alleles were identified, which termed as mixed sites. The mixed sites were identified based on RNA-Seq data as described in^28^. Hybrid offspring from two different species are expected to have higher percentage of mixed sites among all expressed sites than no hybrid species. To create a positive background of hybrid samples, RNA-Seq data of 16 species were pair-wisely mixed and their mixed sites were also identified. The mixed sites of the known hybrid sample F. pringlei* originated from *F. pringlei* × *F. angustiflolia* in^28^ were also identified. The percentage of mixed sites was calculated as the ratio of mixed sites to the total expressed DNA sites in a certain sample.

### Protein divergence, gene expression divergence and morphology divergence

Pair-wise protein divergence (dN) was calculated by applying codeml program in PAML package^58^ by using F3X4 condon frequency. The input super CDS sequence was from the linked coding sequences (CDS) as used in construct phylogenetic tree of *Flaveria* genus^28^, which contains 2,462 genes. Gene expression divergence was calculated as Euclidean distance applying R package based on gene expression values (FPKM) of 12,218 genes. Encoded morphology values of 30 morphology traits were from^16^. The morphology divergence was calculated as Euclidean distance of morphology values. Expression and morphology values were normalized using quantile normalization applying preprocessCore package in R. Linear regression of pair-wise correlation was inferred apply lm function in R package.

### Relative difference of each ancestral node in the phylogenetic tree

The protein sequences at the whole transcriptomic scale of ancestral node were predicted using FASTML^59^. The protein alignment was from^28^. Gene expression abundance and morphological characteristics of all ancestral nodes were predicted by applying ape package of R which uses a maximal likelihood method. For all C_4_ related gene expression, protein sequences and physiological data, their values of the ancestral nodes were assigned to those of the most recent species derived from the node.

Relative difference of protein sequence at each ancestral node was inferred by comparing the sequence at this node (N) with the nearest preceding node of N (N[pre]), *e.g.*, the number of different amino acid between N2 with N1 is the number of changed amino acid at N2. The number of different amino acid changes divided by the aligned length of the protein was calculated as relative protein difference for each gene. Relative difference of gene expression and morphology were calculated as (N -N[pre])/N[pre]. In most cases, the nearest preceding node of N[i] is N[i-1], there are two exceptions: the ancestral node of N11 is N5, and N10 is N8. One-way ANOVA analysis followed by Tukey’s Post Hoc test was used to calculate the significance of relative difference between any two ancestral nodes. *P* values were adjusted by *Benjamin-Hochberg* (BH) correction.

## Supplementary Information

Supplementary Information 1.

Supplementary Information 2.

Supplementary Information 3.

## Data Availability

All data generated during this study are included in this published article and its supplementary information files. Codes used during the current study are available from the corresponding author on reasonable request.

## Abbreviations

*A*: CO2 assimilation rate
AlaAT: Alanine aminotransferase
AspAT5: Aspartate aminotransferase 5
BSCs: Bundle sheath cells
CET: Cyclic electron transport
CRR1: Chlororespiratory reduction 1
DE: Differentially expressed
FPKM: Fragments per kilobase of transcript per million mapped reads
GDC: Glycine decarboxylase complex
GLYK: Glycerate kinase
GOGAT: Glutamine synthetase and glutamine oxoglutarate aminotransferase
GSL1: Glutamine synthetase-like 1
MCs: Mesophyll cells
NADP-ME: NADP-dependent malic enzyme
NCBI: National Center for Biotechnology Information
Ndh: NADH dehydrogenase-like
NHD1: Sodium: hydrogen (Na + /H +) antiporter 1
PEPC: Phospho*enol*pyruvate carboxylase
PGR5-like: Proton gradient regulation 5 like
PIFI: Post-illumina chlorophyll fluorescence increase
PNUE: Instantaneous photosynthetic nitrogen use efficiency
PPCKA: PEPC protein kinase A
PPDK-RP: PPDK regulatory protein
PPDK: Pyruvate, orthophosphate dikinase
PWUE: Instantaneous photosynthetic water use efficiency
SHM: Hydroxymethyltransferase
SRA: Sequence Read Achieve
Γ: CO_2_ compensation point
