# Supplementary material for: The coordination of major events in C4 photosynthesis evolution in the genus Flaveria
Source: Sci Rep. 2021 Aug 2;11:15618. doi: 10.1038/s41598-021-93381-8 (PMC8329263; doi:10.1038/s41598-021-93381-8)
Supplement: Supplementary file 1 — Supplementary Information 1. [file 41598_2021_93381_MOESM1_ESM.docx]

**Supplementary Methods**

### RNA-Seq data retrieval

RNA-Seq data of 14 samples from nine *Flaveria* species, including *F. cronquistii* juvenile leaf (J) and mature leaf (M), *F. angustifolia* J and M, *F pubescens* J and M, *F. sonorensis* J, *F. palmeri* J, *F. vaginata* J, *F. bidentis* J and M, *F. trinervia* J and M, *F. kochiana* M, were downloaded from the Sequence Read Achieve (SRA) of the National Center for Biotechnology Information (NCBI). Growth conditions in greenhouses at the University of Toronto (UT) and sample extraction were carried out as described by Lyu *et al*. [[1](#_ENREF_1)]. These sequences were from an Illumina paired-end sequencing strategy [[1](#_ENREF_1)]. Other plants were grown in greenhouses at Heinrich-Heine University (HHU) [[1](#_ENREF_1)]. RNA-Seq data of these plants were from 13 leaf samples from 13 *Flaveria* species, including *F. robusta, F. angustifolia, F. floridana, F. pubescens*, *F. chloreafolia,* *F. anomala*, *F. ramosissima,* *F. brownii*, *F. palmeri*, *F. vaginata*, *F. bidentis*, *F. trinervia* and *F. australasica*, and were downloaded from SRA. RNA-Seq data from HHU were based on an Illumina single-end sequencing strategy. RNA-Seq data for stem and root samples of *F. robusta* and *F. bidentis* from HHU were generated from the same plants and with the same strategy as the HHU leaf samples. These data were downloaded from SRA. 454 RNA-Seq of 4 *Flaveria* species, including *F. robusta*, *F. ramosissima*, *F. trinervia* and *F. bidentis*, were downloaded from SRA [[2](#_ENREF_2)]. All accession numbers for RNA-Seq data are shown in Table S1. It should be mentioned here that plants in UT and HUU were grown under different conditions (e.g., naturally illuminated greenhouse condition in UT and additional light for 16h per day greenhouse condition in HUU) and samples were taken from different developmental stages (e.g., juvenile samples from UT were taken from the most recently starting to expanding leaves and mature samples were taken from the newly expanding leaves, whereas leaf samples from HUU were taken from plants at 50~60 cm in height before flowering).

### Amino acid sequence extraction and alignment

Predicted amino acid changes between C_3_ and C_4_ species were investigated transcriptome-wide as follows (Fig. S5): (1) Predict peptide sequence from assembled contigs. Both OrfPredictor [[3](#_ENREF_3)] and Portrait [[4](#_ENREF_4)] were applied independently to predict the most possible open reading frames (ORF) in a six-frame way. (2) Filter short protein sequences. Protein sequences from all species were gathered and categorized into two datasets according to their length. Sequences that were no less than 80 % and no more than 120 % of the length of the Arabidopsis reference were classified into dataset 1. Those that were less than 80 % but no less than 50 % of the length of the Arabidopsis reference were classified into dataset 2. (3) Filter out incorrectly assembled sequences from dataset 1. We performed an all against all BLASTP (BLAST+ 2.2.28) [[5](#_ENREF_5)] with an E-value cutoff of 1.0 E-10 in dataset 1. Protein sequences without any similar sequences in dataset 1 were filtered out. The remaining sequences from dataset 1 generated dataset 3. At the same time, the sequence that was targeted as the best hit by the most number of sequences in dataset 1 was used as a reference (RF). (4) Recall next-best sequence from dataset 2 by applying BLASTP [[5](#_ENREF_5)] using RF as reference. Sequences recalled from dataset 2 generated dataset 4. (5) Generate the final protein sequences from each gene by combining dataset 4 and dataset 3, termed as dataset 5. If there were multiple sequences present in dataset 5 for one species, then the one with the highest BLASP bit score with RF was saved and all others discarded. (6) Align sequences in dataset 5 using MUSCLE [[6](#_ENREF_6)]. Finally, the predicted amino acid sequences from the C_4_ species were compared to those of the C_3_ species to identify changes. A difference was classified as a change if the homologues from the two C_3_ species (*F. robusta* and *F. cronquistii*) contained the same predicted amino acid at a position, but was different with the predicted amino acid at the corresponding position in homologues from at least two C_4_ species (among *F. kochiana*, *F. bidentis*, *F. trinervia* and *F. australasica*). An in-house Perl script was used to make the comparisons. It should be mentioned that C_4_ specific mutations identified here may be lineage-specific, and not necessarily related to C_4_ photosynthesis.

### Estimation of accuracy of predicted protein sequences

To estimate the accuracy of the predicted sequences of identified proteins, we conducted a systematic comparison between the sequences of proteins from *Flaveria* species available in the UniProt KnowledgeBase (UniProtKB) database (<http://www.uniprot.org/>) with the corresponding sequences predicted using our pipeline. A total number of 20 protein sequence pairs were compared. For each pair, after aligning with MUSCLE [[6](#_ENREF_6)], the number of equivalent amino acids was calculated.

### Test whether genes from different pathways are significantly enriched in the 56 modified genes

We tested whether genes from C_4_ pathway, cyclic electron transport chain (CET) and photorespiratory pathway are significantly enriched in the 56 modified genes using *Fisher’s* exact test. Based on collected genes list from the three pathways [[2](#_ENREF_2), [7](#_ENREF_7), [8](#_ENREF_8)] (Table S4), we generated tables for *Fisher’s* exact test independently. Take the C_4_ pathway as an example:

we have:

A=16 (total genes in C_4_ pathway)

B=56 (total modified genes)

C=9 (intersection of A and B)

D=12215 (total expressed genes)

| A and B  **(9)** | non-A and B  **(56-9)** |
| --- | --- |
| A and non-B  **(16-9)** | non-A and non-B **(12215-16-56+8)** |

Applying fisher.test function in R, we obtained p < 2.2e-16 and odds ratio=327.9. In total, *Fisher’s* test was performed for three independent times (as we tested three pathways), p value was then normalized using BH method.

**Supplementary Results**

### Investigate the accuracy of predicted peptide sequence comparing with those from UniprotKB

To estimate the accuracy of the predicted peptide sequences from our data, we conducted a comparative study of protein sequences from UniProtKB (http://www.uniprot.org) with those from our data. We obtained all of the *Flaveria* protein sequences available in UniProtKB, 20 of which were assembled as full-length proteins in our dataset. Among these 20 proteins, 14 showed full identity with homologues in UniProtKB (Table S3). Our assembled *F. bidentis* NADP-ME protein sequence showed four amino acids that were inconsistent with the corresponding sequence from UniProtKB, namely N232, V428, G521 and A634 versus I232, A428, S521 and T634 in the UniProtKB entries (Table S3). Considering that the UniProtKB proteins are based on both manual and automatic annotation and consequently may be prone to error, we examined these residues in our assembled sequences from other *Flaveria* species. *F. australasica* and *F. ramosissima* NADP-ME isoforms were predicted to have the same amino acids as the *F. bidentis* NADP-ME generated from our data*,* suggesting that our predicted sequences are correct. A similar situation was also found in three other proteins, *F. ramosissima* maturase K (matK), *F. trinervia* matK and *F. bidentis* PPDK (Table S3). In the case of the *F. brownii* and *F. trinervia* predicted PEPC sequences, we found five and six amino acids, respectively, that were inconsistent between the UniProtKB sequences and our data; however, about half of the amino acids were consistent with sequences from two other species (Table S3). These inconsistencies between our assembled sequences and those in UniProtKB may be a result of assembly artifacts or the presence of multiple alleles in the different *Flaveria* species. Therefore, our predicted peptide sequences are comparable to the sequences from UniProtKB in terms of accuracy.

**Supplementary figures and tables**

**Figure S1. Contig-length distribution of 16 *Flaveria* species**

All species displayed a peak at around 316 nucleotides (10^2.5^).


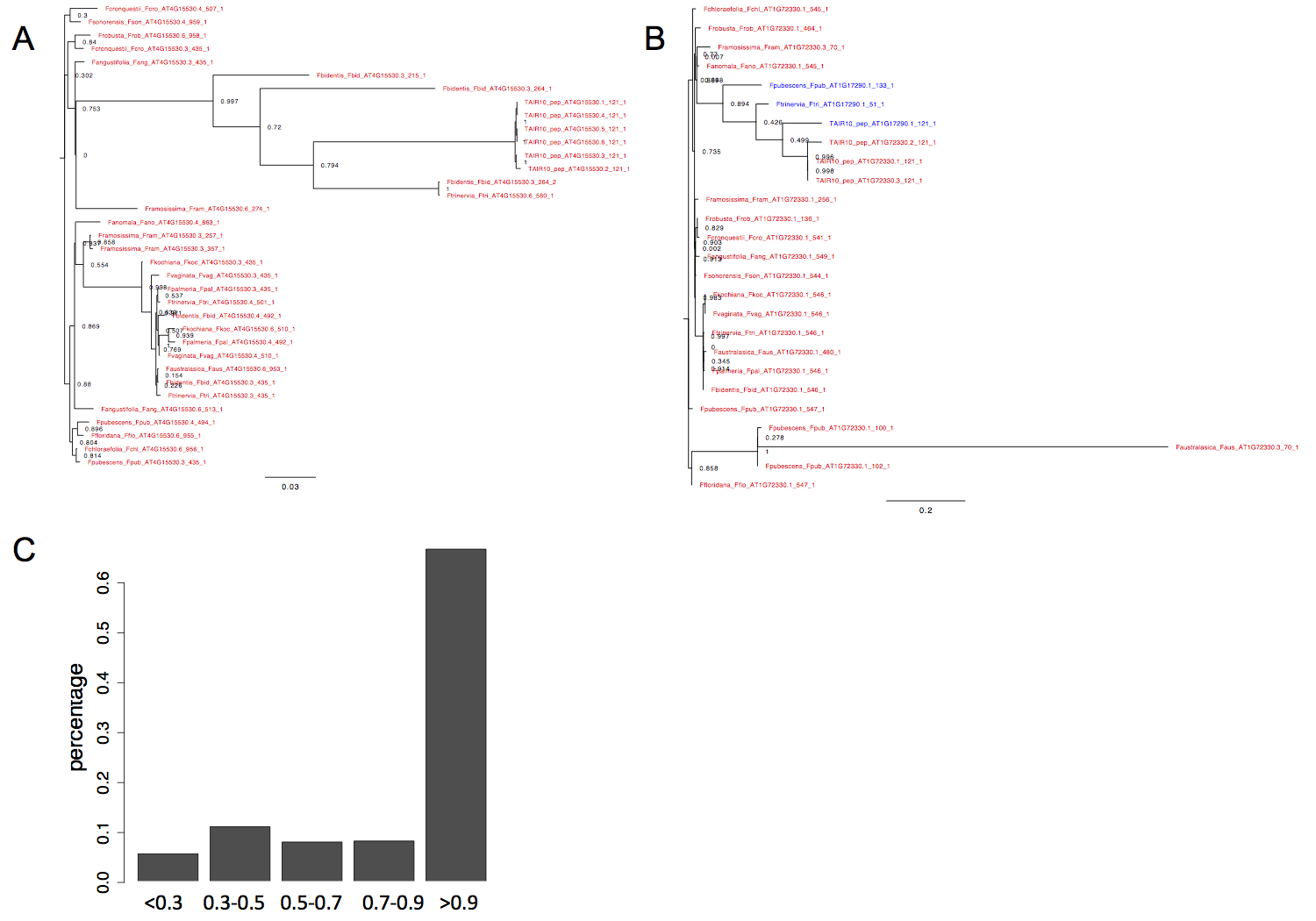


**Figure S2. The consistency between gene annotation based Blast and Orhtologous groups based on OrthoFinder**

A: The consistence between annotation using Blast and orthologous groups was calculated in two categories: (1) If the orthologous group contains Arabidopsis genes, the consistency was calculated as the percentage of genes that have the same annotation with Arabidopsis gene, such as PPDK showed in (A). (2) If the orthologous group contains no Arabidopsis gene, the percentage of each annotated gene ID in this group was calculated, and the highest percentage was assigned as the consistency, such as AlaAT showed in (B). The form of the *Flaveria* gene in the gene trees was as: SpeciesName_SpeciesAbbrevation_Annotation_AssembledProteinLength_SerialNumber. The distribution of the consistency of total 28164 orthologous groups is showed as (C).

**Figure S3. Pair-wise Pearson correlations of 31 *Flaveria* samples.**

Heat map shows transcriptomic profiles from juvenile (j) and mature (m) leaves of the same species showed the highest similarity. In addition, transcriptomic profiles between the same organs from different species were more similar than that between different organs from a same species. The heat map was generated using “heatmap.2” function from the R package gplots (V3.1.1). (Abbreviations: j/m: juvenile/mature leaf sample from University of Toronto, hhu/shoot_hhu/root_hhu: leaf/shoot/root/ samples from Heinrich-Heine-University.)


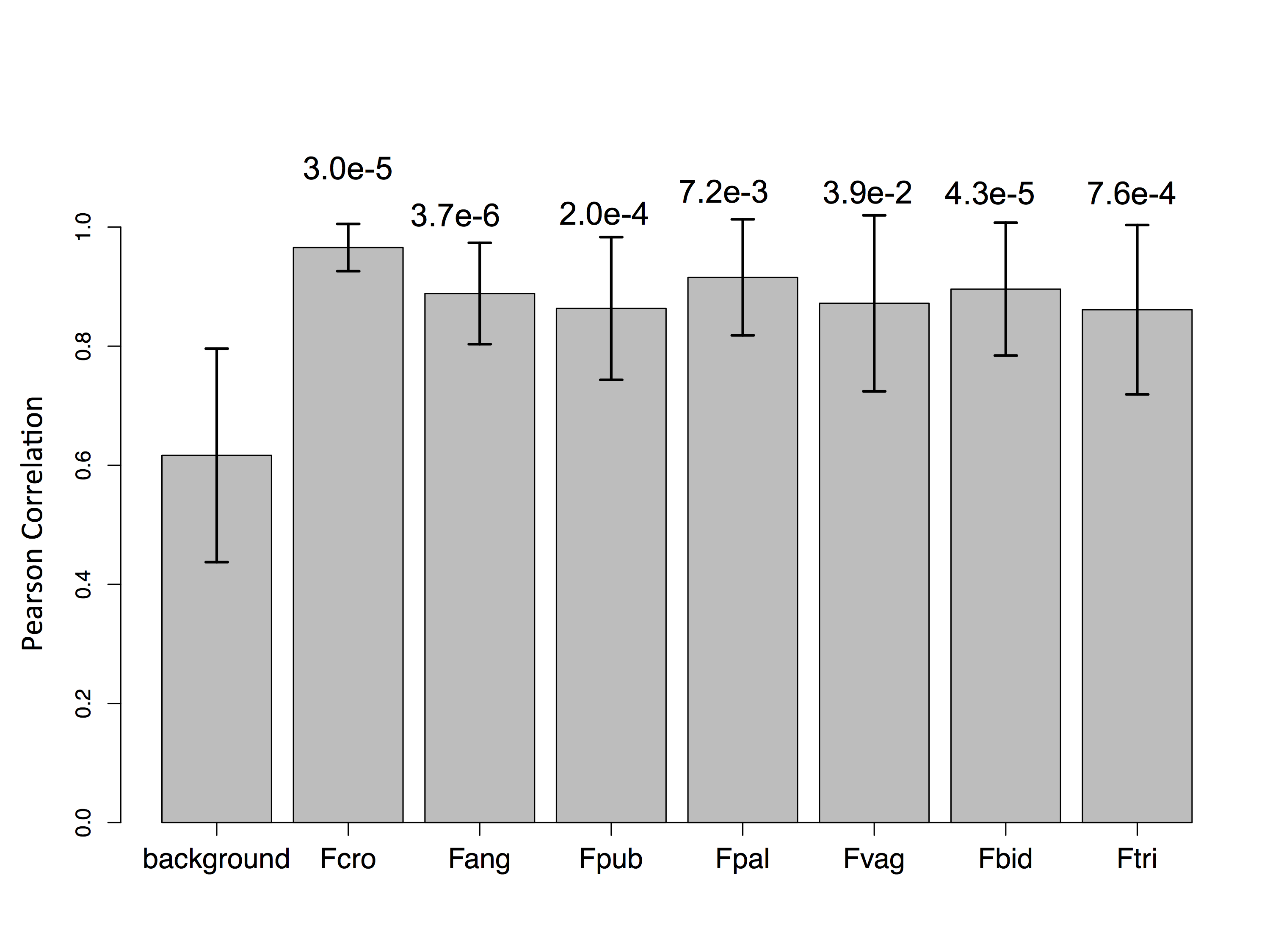


**Figure S4. Pearson correlations between leaf samples from the same species are higher than mean Pearson correlations among leaf samples**

The first bar shows the mean correlations among 29 leaf samples, while the second to the eighth bars show the Pearson correlation between samples of a same species. Error bars show standard deviation. Student tests were conducted to test whether the Pearson correlation within each species is higher than that of background, which was the Pearson correlations of all pair-wise samples, and the P-values are shown at the top of the corresponding bar. Abbreviations: Fcro: *F.cronquistii*, Fang: *F. angustifolia*, Fpub: *F. pubescens*, Fpal: *F. palmeri*, Fvag: *F.vaginata*, F bid: *F. bidentis*, Ftri; *F. trinervia*.


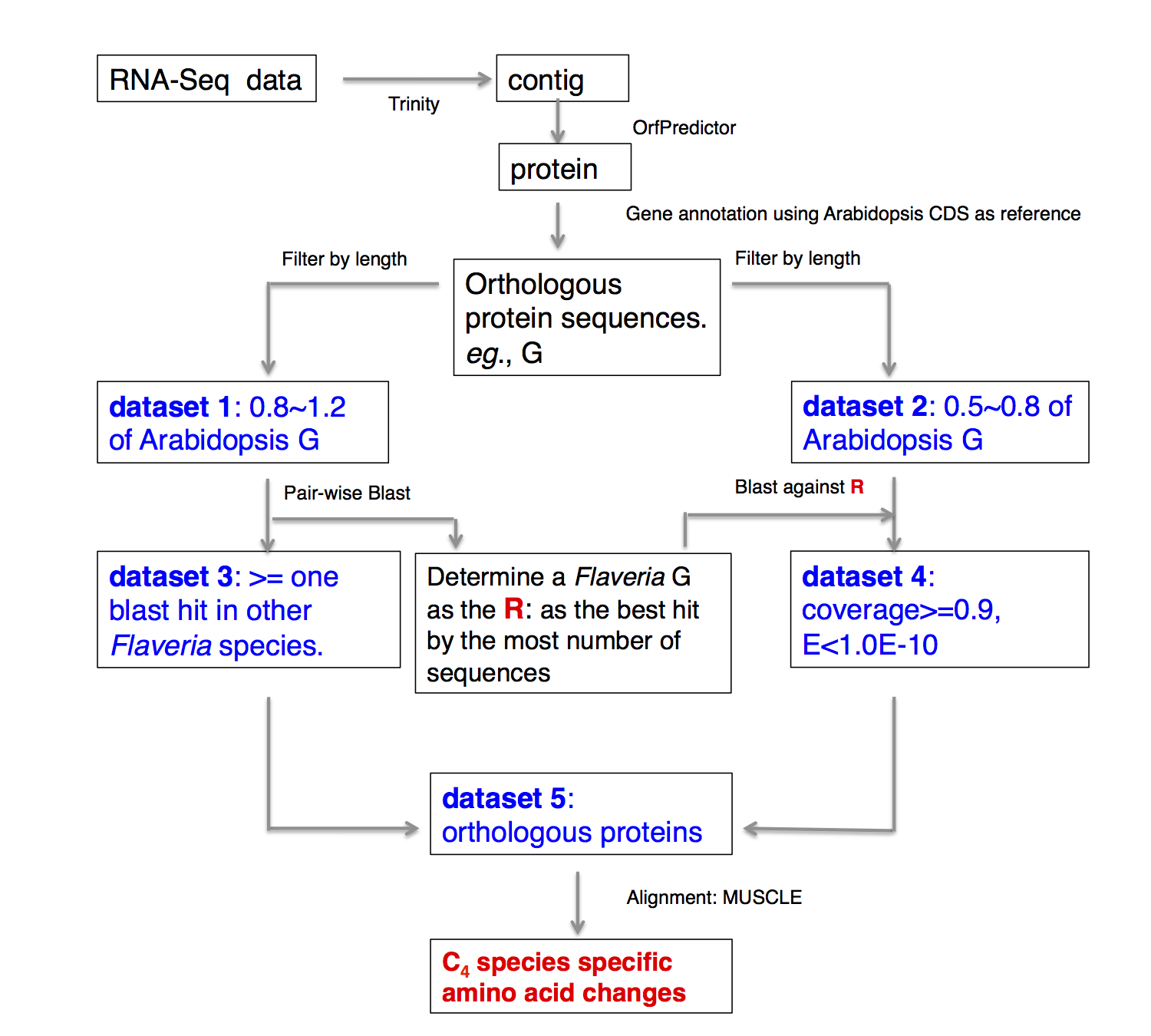


**Figure S5. Workflow to identify amino acid changes between C_3_ and C_4_ species from RNA-Seq data.**

Transcripts were first assembled based on RNA-Seq data. Open reading frame (ORF) and protein sequences were then predicted from assembled transcripts. Proteins were annotated as the best hit of Blast against Arabidopsis CDS reference. Orthologous genes, *eg.*, “G”, from all *Flaveria* species formed the orthologous group of “G”, the orthologous group of “G” were classified into two groups according to their length: dataset 1 contained protein sequences that had similar lengths to “G” in Arabidopsis, and dataset 2 contained relatively short protein sequences which may result of incompletely assembly. By all against all BLASTP among dataset 1, the sequences in dataset 1 that had no similar sequence in dataset 1 were discarded, these sequences may result from mis-annotation or mis-assembly, and the remaining sequence from dataset 1 formed dataset 3. Meanwhile, the protein sequence from dataset 1 that was the best target of the most number of sequences by BLASTP in dataset 1 was chosen as reference (R) to retrieve good sequence from dataset 2 by using BLASTP. This step resulted in dataset 4, which form dataset 5 together with dataset 3. Mutations were determined from alignment of protein sequences in dataset 5, where the sequences of at least two C_4_ species were consistent, but differed from the two C_3_ species.


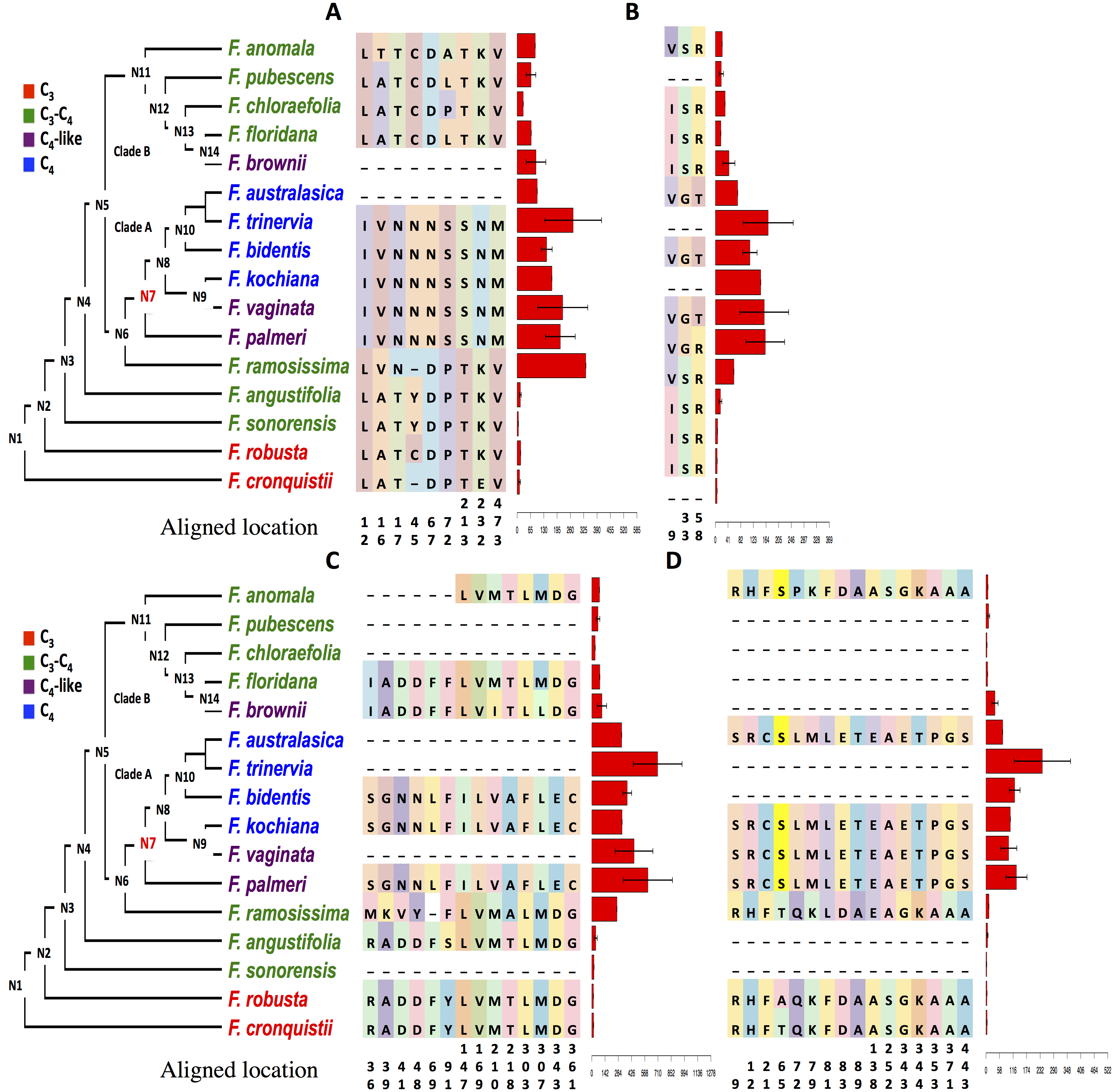


**Figure S6. Predicted changes in amino acid residues and transcript abundances of C_4_-associated aminotransferases and transporters**

The changes in amino acid residues and levels of transcripts (FPKM) encoding alanine aminotransferase (AlaAT) (A), aspartate aminotransferase 5 (AspAT5) (B), sodium symporter family protein 2 (C) and sodium:hydrogen (Na^+^/H^+^) antiporter (D) are shown for C_4_ and C_3_ *Flaveria* species. Only the amino acid residues predicted to be different between C_3_ and C_4_ species are superimposed on the *Flaveria* phylogenetic tree (Lyu et al., 2015). The colors of amino acid residues have no meaning and are only for visualization purposes. Numbers below the amino acids indicate the location site in the multiple sequence alignments. FPKM values are represented to the right of the amino acid changes as red bars.


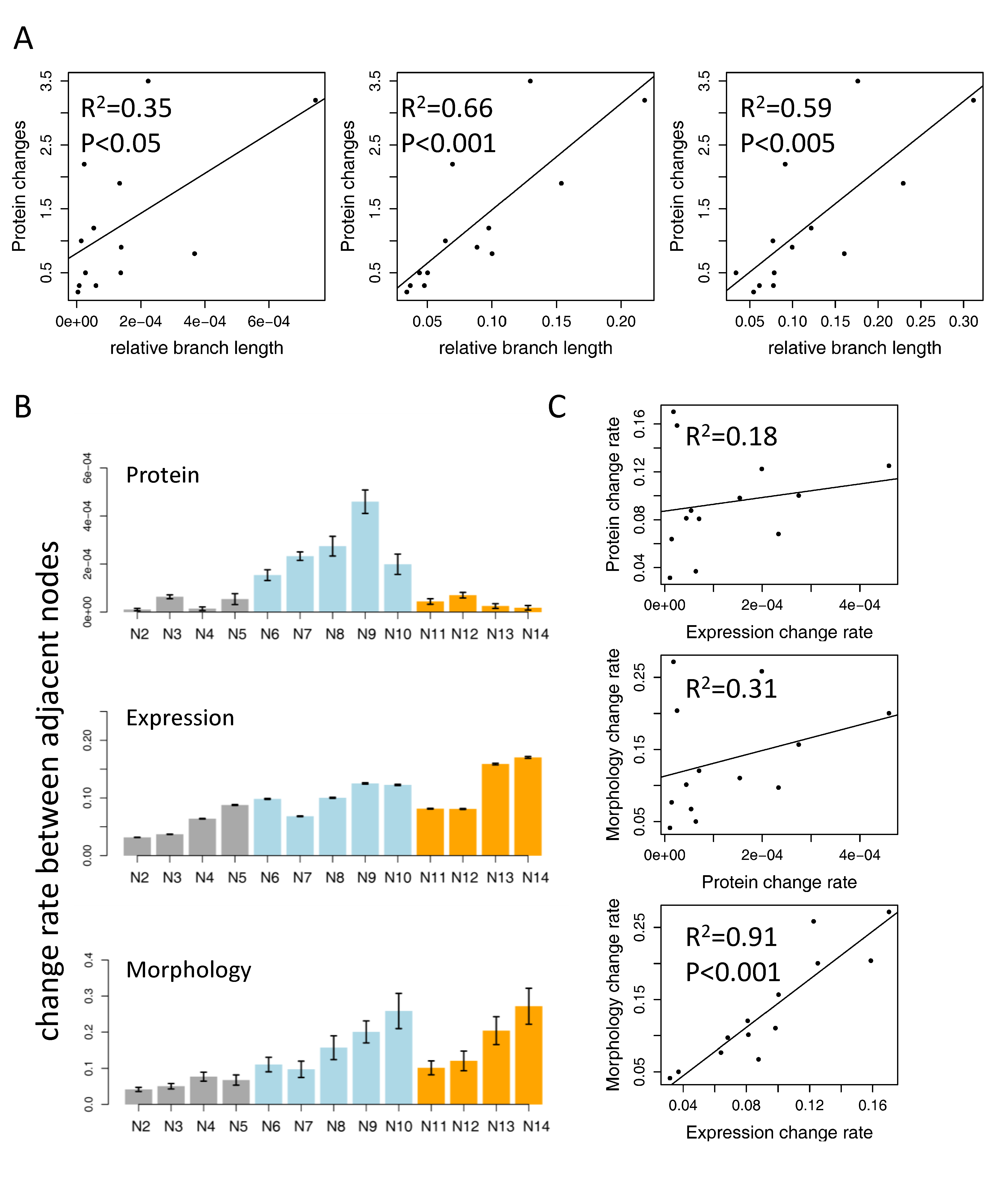


**Figure S7. Change rates of ancestral nodes in protein sequence are not correlated with change rates of gene expression or morphological traits**

The changes between ancestral nodes in protein sequence, gene expression and morphology are plotted to the branch length (A). (B) Bar plots show the branch-length normalized changes of ancestral nodes, which are termed as change rates. (C) The Pearson correlations between change rates of protein sequence, gene expression and morphological traits.

**Table S1. The *Flaveria* samples and RNA-Seq data used in this study**

| C_3_ | Type I C_3_-C_4_ | TypeII C_3_-C_4_ | C_4_-like | C_4_ |
| --- | --- | --- | --- | --- |
| Pair-end RNA-Seq from Illumina HiSeq 2000, read length: 90 nucleotides. Plants were grown at the University of Toronto. Mature leaves were taken as the newest fully expanded leaf, and juvenile leaves were taken as the most recently starting to expand from the main stem. Each sample was pooled from 2-4 plants. Raw data were downloaded from NCBI Short Read Archive (SRA). | | | | |
| *F. cronquistii* (j^a^,m^b^) SRX447412,  SRX447413 | *F. angustifolia* (j^a^, m^b^)  SRX447406,  SRX447407  *F. pubescens* (j^a^,m^b^)  SRX447418,  SRX447419  *F. sonorensis* (j^a^)  SRX447420 |  | *F. palmeria* (j^a^)  SRX447415  *F. vaginata* (j^a^)  SRX447423 | *F. bidenits* (j^a^,m^b^) SRX447408,  SRX447409  *F. trinervia* (j^a^,m^b^)  SRX447421, SRX447422  *F. kochiana* (m^b^) SRX447414 |
| Single-end RNA-Seq from Illumina HiSeq 2000, read length: 100 nucleotides. Plants were grown at Heinrich-Heine University. Leaf samples were taken from 2^nd^ and 4^th^ leaf. Root and shoot samples were taken from corresponding plants. SAR accession number underlined were generated in this study and submitted to NCBI SRA, others were downloaded from NACI SRA. | | | | |
| *F. robusta*(l^a^,r^c^,s^d^)  SRX468650,  SRX794075，  SRX794076， | *F. pubescens*(l^a^)  SRX468638  *F. chloreafolia*(l^a^),  SRX467630  *F. angustifolia*(l^a^),  SRX794138 | *F. anomala*(l^a^)  SRX467620  *F. floridana* (l^a^),  SRX794148  *F. ramosissima* (l^a^)  SRX468646 | *F. brownii*(l^a^)  SRX467625  *F. vaginata*(l^a^)  SRX794153  *F. palmeri* (l^a^)  SRX794162 | *F. bidenits*(l^a^,r^c^,s^d^)  SRX467614,  SRX794053，  SRX794064  *F. trinervia*(l^a^)  SRX468662  *F. australasica*(l^a^)  SRX794172 |
| 454 RNA-Seq from GS FLX, read length: 200 nucleotides. Plants were grown at Heinrich-Heine University. Leaf samples were taken from the second and fourth leaves. | | | | |
| *F. robusta*  SRX052096 |  | *F. ramosissima*  SRX052095 |  | *F. bidentis*  SRX052090  *F. trinervia*  SRX052094 |

*^a^* : juvenile leaf, ^b^: mature leaf, ^c^: root sample, ^d^: shoot sample.

**Table S2. Assembly and mapping statistics of 16 *Flaveria* species**

| Species | #Contig | N50 | %Contig mapping to Arabidopsis | #Unique mapping | #Splitting mapping | Proportion Splitting mapping |
| --- | --- | --- | --- | --- | --- | --- |
| *F. angustifolia^a^* | 34,710 | 914 | 68.15% | 23,036 | 620 | 2.62% |
| *F. anomala^b^* | 48,969 | 1,067 | 46.99% | 21,413 | 1,598 | 6.94% |
| *F. australasica^b^* | 47,577 | 1,168 | 52.02% | 23,698 | 1,050 | 4.24% |
| *F. bidentis^a^* | 42,574 | 1,316 | 58.96% | 24,372 | 728 | 2.90% |
| *F. brownii^a^* | 44,794 | 974 | 58.17% | 24,920 | 1,135 | 4.36% |
| *F. chloraefolia^b^* | 41,203 | 1,208 | 56.56% | 22,280 | 1,025 | 4.40% |
| *F. croqustii^a^* | 42,323 | 1,049 | 62.09% | 25,446 | 832 | 3.17% |
| *F. floridana^b^* | 44,561 | 1,119 | 53.42% | 22,622 | 1,181 | 4.96% |
| *F. kochiana^a^* | 35,534 | 1,100 | 63.98% | 22,165 | 571 | 2.51% |
| *F. palmeri^a^* | 36,931 | 1,015 | 65.53% | 23,583 | 618 | 2.55% |
| *F. pubesens^a^* | 39,741 | 1,158 | 64.77% | 25,051 | 689 | 2.68% |
| *F. ramosissima^b^* | 55,618 | 658 | 38.55% | 19,025 | 2,418 | 11.28% |
| *F. robusta^b^* | 44,807 | 1,181 | 53.46% | 22,739 | 1,217 | 5.08% |
| *F. sonorensis^a^* | 39,286 | 1,016 | 64.24% | 24,568 | 671 | 2.66% |
| *F. trinervia^a^* | 30,698 | 903 | 69.74% | 20,800 | 608 | 2.84% |
| *F. vaginata^a^* | 38,424 | 987 | 65.85% | 24,635 | 666 | 2.63% |
| Mean | 417,34 | 1052.0625 | 58.91% | 23,147 | 976.69 | 4.11% |

^a^: contigs were assembled suing pair-end Illumina RNA-Seq reads. ^b^: contigs were assembled suing single-end Illumina RNA-Seq reads. Splitting mapping: isoforms in *Flaveria* mapping to different reference gene loci in Arabidopsis.

**Table S3. Comparison of 20 protein sequences from UniProtKB with those assembled in this study**

Abbreviations: #: number, aa: amino acid; Ftri: *F. trinervia*; Fbid: *F. bidentis*; Fano*: F. anomala*; Fram: *F. ramosissima*; Fkoc: *F. kochiana*; Fbro: *F. brownii*; Fflo: *F. floridana*; Fchl*: F. chloraefolia*; Fpub: *F. pubescens*.

Table S4. Genes list from C_4_ pathway, cyclic electron transport chain and photorespiratory pathway. Gene showing both changes in gene expression and protein sequence between C_3_ and C_4_ species are labeled in red.

**Reference**

1. Lyu MJ, Gowik U, Kelly S, Covshoff S, Mallmann J, Westhoff P, Hibberd JM, Stata M, Sage RF, Lu H *et al*: **RNA-Seq based phylogeny recapitulates previous phylogeny of the genus Flaveria (Asteraceae) with some modifications**. *BMC evolutionary biology* 2015, **15**(1):116.

2. Gowik U, Brautigam A, Weber KL, Weber AP, Westhoff P: **Evolution of C4 photosynthesis in the genus Flaveria: how many and which genes does it take to make C4?** *Plant Cell* 2011, **23**(6):2087-2105.

3. Min XJ, Butler G, Storms R, Tsang A: **OrfPredictor: predicting protein-coding regions in EST-derived sequences**. *Nucleic acids research* 2005, **33**(Web Server issue):W677-680.

4. Arrial RT, Togawa RC, Brigido Mde M: **Screening non-coding RNAs in transcriptomes from neglected species using PORTRAIT: case study of the pathogenic fungus Paracoccidioides brasiliensis**. *BMC bioinformatics* 2009, **10**:239.

5. Shiryev SA, Papadopoulos JS, Schaffer AA, Agarwala R: **Improved BLAST searches using longer words for protein seeding**. *Bioinformatics* 2007, **23**(21):2949-2951.

6. Edgar RC: **MUSCLE: multiple sequence alignment with high accuracy and high throughput**. *Nucleic acids research* 2004, **32**(5):1792-1797.

7. Peng LW, Yamamoto H, Shikanai T: **Structure and biogenesis of the chloroplast NAD(P)H dehydrogenase complex**. *Biochimica Et Biophysica Acta-Bioenergetics* 2011, **1807**(8):945-953.

8. Mallman J, Heckmann D, Brautigam A, Lercher MJ, Webb APM, Westhoff P, Gowik U: **The role of photorespiration during the evolution of C4 photosynthesis in the genus Flaveria**. *eLife* 2014, **3**:e02478.
